# Current Antioxidant Treatments in Organ Transplantation

**DOI:** 10.1155/2016/8678510

**Published:** 2016-06-15

**Authors:** Shaojun Shi, Feng Xue

**Affiliations:** Department of Liver Surgery and Liver Transplantation Center, Renji Hospital, School of Medicine, Shanghai Jiaotong University, Shanghai 200127, China

## Abstract

Oxidative stress is one of the key mechanisms affecting the outcome throughout the course of organ transplantation. It is widely believed that the redox balance is dysregulated during ischemia and reperfusion (I/R) and causes subsequent oxidative injury, resulting from the formation of reactive oxygen species (ROS). Moreover, in order to alleviate organ shortage, increasing number of grafts is retrieved from fatty, older, and even non-heart-beating donors that are particularly vulnerable to the accumulation of ROS. To improve the viability of grafts and reduce the risk of posttransplant dysfunction, a large number of studies have been done focusing on the antioxidant treatments for the purpose of maintaining the redox balance and thereby protecting the grafts. This review provides an overview of these emerging antioxidant treatments, targeting donor, graft preservation, and recipient as well.

## 1. Introduction

Ischemia/reperfusion injury (IRI) is present in many medical situations, specifically in organ transplantation. This event can lead to immediate and long-term graft dysfunction, such as allograft rejection, delayed graft function (DGF), and even primary nonfunction (PNF) [1]. It is directly associated with endothelial and parenchymal cell injury, increased vascular permeability, inflammatory response, and generation of reactive oxygen species (ROS).

Oxidative stress, known as an imbalance between the generation of ROS and antioxidant defense system, is the disease mechanism most commonly involved in IRI. It has been proven that ischemia initiates the noxious generation of ROS, while the reoxygenation process during reperfusion is responsible for most ROS production, activation of complement system, and inflammatory response [2]. Evidence supports the fact that occlusion of the vascular supply during transplantation results in serious hypoxia among the endothelial cells, which turns into an important source and target of ROS. Mitochondrial dysfunction, neutrophil priming, xanthine oxidase, and NADPH oxidases play a pivotal role in this process [3]. Subsequently, excessive oxidants cause tissue damage and cell death by inducing the peroxidation of DNA, protein, and lipids.

The ROS-induced injurious effect on graft and recipient is related to various posttransplant complications. Besides, marginal grafts are significantly vulnerable to the oxidative stress and thus restrict graft pool, which aggravates organ shortage. To alleviate such adverse outcomes, a volume of preclinical and clinical studies against oxidative stress are under investigation, targeting consecutive process throughout the transplantation, including donor, graft preservation, and recipient as well. Despite some new discoveries in the mechanism against ROS, the clinical results remain controversial. Therefore, we summarize some advanced development of antioxidant treatments in organ transplantation and their corresponding mechanisms.

## 2. Antioxidant Treatment for Donor

### 2.1. Local Ischemic Preconditioning

Local ischemic preconditioning (LIPC), a widely accepted antioxidant approach, is a brief period of ischemia/reperfusion that leads to tolerance of subsequent ischemia/reperfusion injury (IRI). In animal studies, LIPC has been proven to be an efficient tool to protect most organs (e.g., liver, kidney, heart, and intestine), particularly via antioxidant pathway [4]. Concerning mechanism, a recent study demonstrates that LIPC can enhance a series of antioxidant genes' expression via activating cytoplasmic redox-sensitive transcription factors, effecting regulating cell redox status, inhibiting oxidative injury to cell components, altering the disturbance of Ca^2+^, and preventing nucleus injury and DNA fragmentation [5]. From bench to bedside, a randomized clinical trial (RCT) involves 60 liver donors by 10 min inflow occlusion and 10 min reperfusion to improve the graft tolerance to IRI, and the results indicate that LIPC approach significantly improves liver biochemical markers of hepatocyte function in deceased donor liver transplantation (LT) [6]. However, another similar clinical study with smaller sample size has not shown any difference between LIPC and control group [7] nor has any beneficial result of LIPC been seen in liver resection surgery [8]. These controversial results may arise from invasive procedure to induce LIPC, in which direct vessel clamping may cause uncontrollable degree of graft injury and thus contribute to varied clinical results. Ischemia duration is another key point. An RCT investigating the safety and efficiency of 5 min LIPC displays that, different from 10 min occlusion, 5 min inflow occlusion does not protect liver grafts from subsequent IRI [9]. To further investigate the effects of time-dependent LIPC, an LIPC model with 5/8/10/15 min inflow occlusion and 10 min reperfusion is performed on rat fatty livers, followed by index ischemia. The result indicates that 5/8 min inflow occlusion is an optimal regimen for protection of fatty liver, with a dramatically lower serous malondialdehyde (MDA) concentration [10]. Moreover, ischemia tolerance differs from organ to organ and clinical studies focusing on other solid organs should also be explored to verify LIPC application, and patient-related factors such as age, gender, or comorbidities should also be taken into consideration.

### 2.2. Hydrogen Preconditioning

Hydrogen is a reducing gas that displays antioxidant properties and exhibits protective effects against graft IRI and dysfunctions. Though application of mechanical ventilation (MV) in intensive care unit supports donor's life, it can also provoke oxidative stress and inflammatory response, causing ventilator-induced lung injury (VILI) and reducing graft viability [11]. To prevent VILI occurrence, inhaled hydrogen, an ROS scavenger, has been applied in a recent study and effectively reduced VILI-associated inflammatory responses, at both local and systemic levels [12]. In lung transplantation, preloading hydrogen in lung tissue decreases toxic ROS during reperfusion [13]. Moreover, hydrogen inhalation at 2% concentration 1 h prior to liver procurement can also protect the liver from IRI by activation of NF-*κ*B signaling pathway [14]. Therefore, hydrogen preconditioning might be a practical way for the treatment of transplant donors, especially for those subjected to MV supporting before graft procurement. There is no consensus on what the most optimal concentration of inhaled hydrogen should be yet. It is reported that concentration of inhaled hydrogen ought to be monitored and kept less than 4.6% when mixed with air and less than 4.1% when mixed with oxygen [15], though the safety of hydrogen inhalation is still needed to be testified clinically. Besides, hydrogen-rich water (HRW) has been developed recently and applied for various clinical purposes. To investigate HRW effects on patients with either type 2 diabetic mellitus or impaired glucose tolerance, an RCT has been performed and shows that hydrogen reduces the concentrations of oxidized low density lipoprotein and free fatty acids, while it increases plasma levels of extracellular-superoxide dismutase (SOD) in diabetics [16]. Another human study shows that HRW remarkably attenuates oxidative stress, improves liver function, and reduces HBV DNA in patients with chronic hepatitis B [17]. Nowadays, some groups are dedicated in developing practical techniques to stabilize hydrogen-rich solution for graft storage during transportation.

### 2.3. Antioxidant Carriers Therapy

High levels of ROS play a pivotal role in transplant-associated IRI, especially in cadaveric donors. Antioxidant enzymes (AOEs), such as catalase and SOD that detoxify H_2_O_2_ and superoxide, are highly potent and specific agents to the ROS-induced injury and not consumed in reaction with ROS. However, due to inability to cross cell membrane barriers and fast elimination, a significant hurdle in the clinical translation lies in the insufficient delivery of these enzymes to targeted sites, especially the vascular endothelium suffering oxidative injury [18]. It is also believed that megadoses of nonenzymatic antioxidants (e.g., N-acetylcysteine (NAC) and curcumin) can only alleviate subtle chronic oxidative stress, whereas their protective effects in acute conditions including IRI and inflammation are extremely limited [19]. To enhance the bioavailability and efficacy of AOEs and nonenzymatic antioxidants, various antioxidant carriers have been developed to protect antioxidant cargoes from inactivation and improve intracellular delivery.

Application of vascular immune targeting AOEs to specific endothelial epitopes has been proven to be an effective donor preconditioning method in lung transplantation model. The nanosized conjugates, consisting of AOEs and specific antibodies, can be directed against the endothelial determinant accumulation in vascular endothelium after intravenous administration and eventually delivered into endothelial cell, thus alleviating oxidative stress. Platelet/endothelial cell adhesion molecule-1 (PECAM-1) is such an endothelial epitope and the specific nanosized particles (anti-PECAM/catalase conjugates) have been examined in a porcine lung transplantation model, in which the immune targeting treatment allows immediate reconstitution of pulmonary gas exchange and microcirculation, and improve both graft and recipient outcomes [20]. Moreover, anti-PECAM/SOD conjugates have also been conformed to specifically downgrade pulmonary endothelial ROS flux [21]. Likewise, angiotensin-converting enzyme (ACE) is another ideal endothelial epitope for immune targeting therapy. Conjugates of ACE monoclonal antibody 9B9 with catalase (9B9-catalase) have been proven to augment antioxidant defenses of pulmonary endothelium in a rat lung IRI model [22]. Despite strong antioxidant effects, the adverse effects induced by immune targeting conjugates, including disturbance of vasoreactivity and pulmonary arteriolar constriction, cannot be neglected in clinical translation process. It should also be considered that immune targeting therapy along with other novel approaches, such as supplementation of NO, targeting of antithrombotic drugs, and donor preconditioning, may be a promising alternative.

Liposomes are artificial vesicles consisting of one or more phospholipid layers, with an aqueous core enclosed; lipid-soluble antioxidants can be incorporated into the lipid bilayer, while water-soluble antioxidants (e.g., NAC and curcumin) can be encapsulated in the aqueous space. NAC is a well-known antioxidant which can function both as a ROS scavenger and as a precursor of reduced glutathione, thus modulating the redox status. To improve the bioavailability, NAC is encapsulated in the aqueous space of liposome, gaining a higher protective potency than the free drug. Liposomal NAC has been administered separately in rodents to protect lung [23] and liver [24] injury and provided a protective effect through antioxidant pathway. Similarly, liposome can be incorporated with a potent inhibitor of the NF-*κ*B pathway, curcumin, to target delivery to renal tubular epithelial and antigen-presenting cells. In a renal IR model, liposomal curcumin provided cytoprotection effect through multiple antioxidant mechanisms following renal IR injury [25].

Conclusively, immune targeting therapy and liposome formulation are promising approaches to facilitate the delivery of AOEs and nonenzymatic antioxidants before ischemia process. Though there is no associated animal transplantation study performed, we hold that the carriers can help the antioxidants administered to a donor quickly bind to endothelium and subsequently protect graft from IRI. However, these carriers must be carefully tested in terms of toxicity, activation of defense systems, inflammation or thrombosis, and aggregation in circulation and embolism of the microvasculature.

## 3. Antioxidant Treatment for Grafts

Ex vivo graft preservation is necessary for allocating and transporting the graft to its recipient. It has been widely accepted that primary occurrence of ROS-induced injury arises from the cellular alteration during the ischemia process, especially in cold preservation period. Several approaches including machine perfusion and modified preservation solutions have been developed to reduce the injury.

### 3.1. Machine Perfusion

Machine perfusion (MP) is increasingly used as an alternative method to overcome the present shortage of donors by expanding the graft pool and prolong the storage time. It is a dynamic technique using a continuous flow of solutions to perfuse and maintain residual metabolism of the graft [26]. In recent years, the interest in MP preservation has been revived, especially in hypothermic machine perfusion (HMP). HMP is able to maintain the viability of non-heart-beating donors (NHBD) grafts effectively, though the HMP-induced cold damage, such as ROS injury, should not be neglected. MP temperature is a key factor affecting antioxidant potential. The ROS production in steatotic liver submitted to MP at 20°C, known as subnormothermic machine perfusion (SNMP), is significantly lower than that of HMP at 8°C or 4°C [27]. Similarly, various clinical studies focusing on normothermic machine perfusion (NMP) have also been carried out. To examine the feasibility of transplanting high-risk donor lungs that has undergone NMP, a prospective clinical trial subjects lungs of high-risk donors to 4 hours of NMP and demonstrates that NMP group shows similar physiological stability to those in control group [28]. In addition, red cell-based perfusate and a short period of oxygen supply during MP are designed to reduce the likelihood of inflammation and oxidative injury. However, few studies have been performed to explore the oxidative stress mechanism in NMP and the pragmatic antioxidant potential of NMP needs to be examined. To further improve the viability of perfused grafts, perfusion mediums supplemented with various antioxidant agents can also alleviate ROS-induced injury effectively [29].

### 3.2. Polymer Solutions

Polyethylene glycol (PEG), synthesized as liner or branched polymers in different sizes, functions as an alternation of hydroxyethyl starch (HES) contained in UW solution due to its low viscosity. As an “immunocamouflage” agent, PEG binds covalently to various biological surfaces and forms complexes with cell membrane lipids, membrane proteins, or carbohydrates, preventing osmotic swelling as well as lipid peroxidation (LPO) in graft cold storage. PEG is also an effective free radical scavenger and can modulate oxidative stress during preservation. Owing to these protective effects, several PEG-based preservation solutions, including Polysol, IGL-1 solution, and SCOT, have been developed for organ preservation.

Polysol solution is a colloid-based low-viscosity organ preservation solution containing vitamins, amino acid, and a variety of ROS scavengers (including allopurinol, glutathione, alpha-tocopherol, and ascorbic acid), which possess strong antioxidant capacity. Polysol solution is applied on a steatotic rat liver perfusion model and significantly attenuates LPO to nearly one fourth of that in HTK control [30]. In rat partial liver transplantation, Polysol solution also brings a protective effect on overall quality of partial liver graft, evidenced by improved microcirculation, higher graft compliance, less hepatocyte damage, reduced apoptosis, and improved regeneration [31]. However, to assess the safety of Polysol solution for clinical application, a human study engages nine donor-recipient couples in adult living kidney transplantation and uses Polysol or UW solution for washout and cold storage of kidney grafts, respectively. The result demonstrates a high incidence of acute rejection and antibody-mediated rejection episodes in the recipients of Polysol solution group [32]. It is obvious that the complex composition of Polysol solution does not permit an accurate elucidation of the mechanisms in terms of both protective and detrimental effects.

Institute Georges Lopez-1 (IGL-1) solution is characterized by lower viscosity (1.250 mm^2^/s), higher sodium, and lower potassium compared with UW solution. The application of IGL-1 solution has been reported in the SCS of pancreas [33], kidney [34], intestine [35], and liver [36]. IGL-1 solution can inhibit endothelial dysfunction and protect graft against oxidative stress through activation of eNOS by both AMPK and AKT pathway. Livers preserved in IGL-1 solution are better protected from IRI than those in Celsior solution, with reduced liver injury, improved function, and less oxidative stress [37]. A recent human study randomly assigns deceased donor liver grafts to IGL-1 or UW solution for preservation and subsequent implantation. Until postoperative day 30, the incidence of hepatic artery thrombosis, PNF, and biliary nonanastomotic strictures are similar in both groups, while the costs of preservation solution for one liver procurement are 992.0 Euros for IGL-1 solution versus 1609.0 Euros for UW solution [36]. Obviously, IGL-1 solution exhibits comparable efficacy and safety to those of the reference preservation solutions, with a lower cost as well.

The Solution de Conservation des Organes et des Tissus (SCOT) has shown its protective potential in a pancreatic islet transplantation model, reducing IRI and ameliorating the long-term outcome of recipients' immune response [38]. To examine the safety of SCOT, a clinical trial performs 29 kidney transplantations (25 cadaveric donors and 4 living related donors) and applies SCOT for in situ washout and SCS. In the first 3 months after surgery, kidney function of SCOT group is comparable to that of controlled UW solution group, which verifies preliminary safety and efficacy of SCOT [39]. However, the long-term outcome of recipients should be monitored further. In addition, to facilitate clinical application, the concentration and chain length of PEG in SCOT is examined on an islet transplantation model. The SCOT containing PEG 20 kDa 15 g/L significantly prolongs allograft survival and induces no PNF and DGF, and thus it may be the most optimal concentration and chain length of PEG for islet graft preservation [38].

### 3.3. Gaseous Supplements

Hydrogen-rich preservation solution has been proven to have high antioxidant potential and tested in liver, kidney, pancreas, bone marrow, lung, and intestinal cold storage [40]. The antioxidant property of hydrogen-rich preservation solution might arise from inhibition of high mobility group box 1 (HMGB1) release and ROS scavenging effect [41]. A novel hydrogen-rich UW solution (HRUW) has been tested for cold preservation and subsequent renal transplantation in rats, showing that HRUW solution can improve renal function and prolong rat survival rate by protecting tubular epithelial cells from inflammation and apoptosis [42]. In rat intestinal transplantation, HRUW can reduce graft damage and protect the recipient from the systemic effects of transplantation via alleviating graft oxidative stress, ultimately facilitating recipient survival [43]. Moreover, the combination of hydrogen inhalation for donor and the hydrogen-rich preservation solution for graft is a prospective way to prolong the graft preservation time and the survival of recipient, which requires further basic and clinical studies.

NO is a kind of free radical diatomic gas and gaseous signaling molecule. The protective potential of NO is associated with the reduction of superoxide anion-induced tissue toxicity and the inflammatory response. Furthermore, NO can modulate mitochondrial energy generation and thus decrease ROS formation during I/R period. Kageyama et al. [44] investigated the effect of venous systemic oxygen persufflation (VSOP) supplemented with NO gas during cold storage of liver grafts and demonstrated that NO combined with VSOP could recondition warm ischemia-damaged grafts, presumably by decreasing ET-1 upregulation and oxidative damage. Similarly, ventilation of NHBD lung grafts with NO during warm ischemia, ex vivo perfusion, and posttransplantation can also reduce IRI and ameliorate lung injury [45]. Although NO has been used as a clinical therapy for pediatric acute respiratory distress syndrome at present, the application of NO in clinical organ preservation is still under investigation, possibly owing to the lack of the safety studies.

Carbon monoxide (CO) is also a gaseous signaling molecule and possesses a high affinity for heme prosthetic group. CO supplemented to preservation solution has been proven to improve the graft function in experimental studies [46, 47]. A preclinical study performs ex vivo delivery of CO to rat kidney graft and suggests that CO remarkably reduces oxidative injury and improves recipients' survival compared to the control group. The combination of CO and cytochrome P450 (CYPs) may interpret the potential mechanism, which reduces ROS production via blocking CYPs degradation and harmful heme/iron release [48].

Hydrogen sulfide (H_2_S) is considered as the third gaseous signaling molecule with properties to help relax vascular smooth muscle, inhibit apoptosis, modulate inflammatory response, and alleviate oxidative stress [49]. NaHS (a source H_2_S) has been proven to possess better antioxidant potential in vitro when compared to that of L-arginine (a source of endogenous NO). The effective doses of both compounds in human body are believed to be 56 *μ*M for H_2_S and 1.2 g/mL for L-arginine, respectively, which provides essential information for subsequent human study [50].

### 3.4. Pharmacologic Approaches

One of the major sources of ROS is the mitochondria that are particularly vulnerable to oxidative injury. Mitochondrial damage may impair the electron flow and promote the formation of superoxide. Mitochondrial permeability transition (MPT) plays an essential role in cell death during IRI, induced by ROS and reversely aggravating the oxidative stress [51]. To protect mitochondrial integrity during ischemia, various antioxidant agents have been developed and applied to preclinical studies. Melatonin, an essential ROS scavenger, is an inducible nitric oxide synthase (iNOS) inhibitor and well-known antioxidant substance secreted from pineal gland, which can reduce mitochondrial swelling before I/R occurrence. Treated by melatonin supplemented IGL-1 solution, the liver grafts with/without steatosis are protected from IRI, possibly owing to increased NO generation (via constitutive e-NOS activation) and reduction of oxidative stress [52].

Ascorbic acid (AA) is a potent physiological extracellular scavenger of ROS. AA has been supplemented into HTK and Polysol solution to prevent ROS. Noticeably, high-concentrated AA has been reported to aggravate hepatic IRI owing to its excess reduction of iron [53]. Alpha-tocopherol (Vit E), an exogenous antioxidant, prevents the process of LPO in both cell membranes and plasma lipoproteins. Trolox is a water-soluble analogue of Vit E and provides similar antioxidant property to that of Vit E. In a porcine heart transplantation model, trolox-UW perfusion has shown remarkable antioxidant effect against IRI [54]. Matrix metalloproteinases (MMPs) are associated with oxidative stress in cardiovascular diseases. Doxycycline (DOX), an antibiotic of tetracycline family, has been found to inhibit MMP-2 expression and thus protects cardiac function from IRI [55]. The cardioplegia solution containing DOX shows protective effects in heart preservation, despite its potent role in modulating cellular redox status during SCS [29].

## 4. Antioxidant Treatment on Recipients

### 4.1. Ischemic Postconditioning

Remote ischemic postconditioning (RIPoC) is induced by several cycles of I/R on a remote tissue (arm or leg) to produce systemic protection against IRI in distant organs, without direct access to the vessels of the organ of interest. RIPoC can increase antioxidant capacity of liver and kidney temporarily by reducing NF-*κ*Bp65 and MDA expression, as well as ameliorating the destructive capacity of oxygen free radicals. From bench to bedside, induced by three cycles of brief 5 min repetitive ischemia and reperfusion via clamping the exposed external iliac artery, Wu et al. [56] demonstrated that RIPoC could enhance the early renal function recovery in renal recipients after transplantation. However, Kim et al. [57, 58] performed two RCTs including living and kidney transplantation separately. The results display that RIPoC did not improve graft function after operation, possibly due to different RIPoC protocols with an arterial tourniquet cuff placed around the patients' thigh or direct occlusion of the macrovessel, whereas RIPoC appeared to be feasible and safe [59]. Although the potential role of RIPoC in transplantation remains to be determined, this approach provides a simple intervention to protect all the grafts against IRI, particularly via the antioxidant pathway. Noticeably, this approach requires predictable timing of organ donation and may not be applied for DCD donors. In addition, a meta-analysis has pointed out that RIPoC stimulus should be delivered 24 h before the index ischemia [60]. Nevertheless, there is no consensus on how many ischemic stimuli should be performed and what the duration of the transient I/R periods should be, which leads to varying results in basic and clinical studies. Formulating a well-accepted protocol for specific organ, as well as clarifying the potential mechanism, is needed in the translating process.

Similar to LIPC, local ischemic postconditioning (LIPoC) is defined as rapid and intermittent interruptions of blood flow in the early phase of reperfusion after a prolonged period of ischemia. In a canine autotransplantation model, after flushing and static preservation of the kidney for 24 hours, LIPoC was performed with six cycles of 10 or 30 seconds or three cycles of 1-minute I/R before final reperfusion. The result indicates enhanced level of expression of SOD and decreased levels of MDA, implying that LIPoC may protect the graft via an antioxidant pathway [61], whereas another clinical study displays that LIPoC is feasible and appeared safe in human DCD renal transplantation, though without any better renal function observed, which requires further investigation [62].

## 5. Conclusion

Oxidative stress is a common cause of PNF, DGF, and allograft rejection, especially in marginal donors. This review summarizes the innovative antioxidant treatments for the donor, graft preservation, or recipient designed to improve the graft viability and long-term outcome (Tables 1(a), 1(b), and 1(c)). However, the lack of intensive studies concerning the mechanism of oxidative stress hampers the development of these approaches. Ischemia conditioning (LIPC, RIPoC, and LIPoC) is believed to be a series of prospective approaches regardless of its controversial benefit and complex procedure. Considering the safety and effectiveness, the consensus of the protocol should be made on the basis of experimental and clinical trials.

Although SCS has been effective for decades on optimal organs preservation, the preservation protocol is not adapted to the increasing marginal grafts which is able to extend the graft pool. Thus, the novel antioxidant preservation solution, as well as various supplements, requires more mechanism researches and pragmatic RCT. Combined utilization of antioxidant approaches may be more promising than attempts to reinforce the antioxidant capacity of organs by a single agent functioning as ROS scavenger. Even though the antioxidant potential of NMP is still unclear, this approach is worth more intensive researches. To this end, though a combination of antioxidant treatments seem to provide the best outcome, accurate models for preclinical studies and unified protocols for clinical trials are needed before the treatments can be translated into clinical practical. Moreover, the potential adverse effect of local and systemic antioxidant interventions to donors, grafts, and recipients, such as host defense hazard and prooxidant effects, should not be neglected. Antioxidant administration should also be controlled at a gradual, controlled rate, thus avoiding burst release and comprising effect.

## Figures and Tables

**(a):** 

Treatment	Subject	Organ	Model or disease	Effects
*Local ischemic preconditioning*	Rat [5]	Liver	I/R	NF-*κ*B ↓, MDA ↓, MPO ↓, AST ↓, ALT ↓, Proinflammatory cytokines ↓
	Human [6]	Liver	LiT	Apoptosis ↓, PNF ↓, AST ↑, HIF-1*α* ↓
	Human [7]	Liver	LiT	No beneficial effect
	Human [8]	Liver	LiR	No beneficial effect
	Human [9]	Liver	LiT	10 min occlusion is optimal
	Rat [10]	Liver	I/R	5/8 min occlusion is optimal

*Hydrogen preconditioning*	Mice [12]	Lung	MV	W/D ratio ↓, MDA ↓, Egr-1 ↓, TNF-*α* ↓, IL-1*β* ↓, CCL2 ↓, apoptosis ↓
	Rat [13]	Lung	LuT	PO_2_ ↑, PCO_2_ ↓, ICAM-1 ↓, IL-1*β* ↓, IL-6 ↓, MDA ↓
	Rat [14]	Liver	I/R	NF-*κ*B ↑, HO-1 ↑, Bcl-2 ↑
	Human [16]	Diabetic	T2DM	LDL ↓, SOD ↑
	Human [17]	Liver	HBV	HBV DNA ↓, ALT ↓, TBiL ↓, SOD ↑, GST ↑

*Antioxidant carriers therapy*				
Immune targeting therapy	Pig [20]	Lung	LuT	Gas exchange ↑, W/D ratio ↓, Edema ↓, MDA ↓
	Human [21]	Cell	HUVECs	VCAM ↑, TNF ↓, IL-1*β* ↓, LP S ↓
Liposome	Rat [22]	Lung	I/R	PO_2_ ↑, endothelin-1 ↓, iNOS ↓
	Rat [23]	Liver	LPS-LiI	NPSH ↓, MDA ↓, 4-HNE ↓, ALT ↓, AST ↓, TNF-*α* ↓
	Rat [24]	Lung	LPS-LuI	NPSH ↓, MDA ↓, 4-HNE ↓, MPO ↓, TNF-*α* ↓

**(b):** 

Treatment	Subject	Organ	Model or disease	Effects
*Machine perfusion*	Rat [27]	Liver	HMP	MP at 20°C is optimal, AST ↓, LDH ↓, ATP/ADP ↑, bile production ↑, TNF-*α* ↓
	Human [28]	Lung	LuT	Subtle beneficial effect
	Rat [29]	Heart	HMP	Apoptosis ↓, MMP-2 ↓, H_2_O_2_ ↓, pAkt/Akt ↑

*Polymer solutions*				
Polysol solution	Rat [30]	Liver	SCS	AST ↓, GLDH ↓, PVP ↓, ATP ↑, O_2_ consumption ↑, bile production ↑, MDA ↓, W/D ratio ↓
	Rat [31]	Liver	PLiT	PVF ↑, ALT ↓, LDH ↓, MDA ↓, VEGF ↑
	Human [32]	Kidney	KT	Acute rejection rate ↑
IGL-1 solution	Pig [33]	Pancreas	PT	Same degree of safety and effectiveness with UW solution
	Human [34]	Kidney	KT	DGF ↓, Cr ↓, apoptosis ↓, Ccr ↑
	Pig [35]	Intestine	IAT	Acute cellular rejection ↓, iNOS ↑, necrosis ↓, apoptosis ↑,
	Human [36]	Liver	LiT	Same degree of safety and effectiveness with UW solution
SCOT solution	Mice [38]	Pancreas	PT	PNF + DGF + allograft survival time ↑
	Human [39]	Kidney	KT	Same degree of safety and effectiveness with UW solution

*Gaseous supplements*				
Hydrogen	Rat [41]	Liver	I/R	ALT ↓, HMGB1 ↓, MDA ↓, TNF-*α* ↓, IL-6 ↓
	Rat [42]	Kidney	KT	Recipient survival rate ↑, Cr ↓, Ccr ↑, MDA ↓, 8-OHdG ↓
	Rat [43]	Intestine	IAT	MDA ↓, LDH ↓, EGR-1 ↓, IL-6 ↓, iNOS ↓, IL-1*β* ↓
Nitric oxide	Rat [44]	Liver	LiT	ALT ↓, HA ↓, MDA ↓, eNOS ↑, iNOS ↓, ET-1 ↓, 8-OHdG ↓
	Rat [45]	Lung	LuT	W/D ratio ↓, vascular resistance ↓, cGMP ↑, iNOS ↓, TNF-*α* ↓
Carbon monoxide	Rat [46]	Kidney	KT	Recipient survival ↑, IL-6 ↓, TNF-*α* ↓, iNOS ↓, PARP ↑
	Rat [48]	Kidney	KT	ALAS-1 ↑, MDA ↓, IL-6 ↓, TNF-*α* ↓, Egr-1 ↓, Cox-2 ↓, Ccr ↑

*Pharmacologic approaches*				
Melatonin	Rat [52]	Liver	SCS	AST ↓, ALT ↓, BSP clearance ↑, vascular resistance ↓, eNOS ↑, TNF-*α* ↓, MDA ↓, HO-1 ↑
Trolox	Pig [54]	Heart	HT	ET-1 ↓, MDA ↓, SOD ↑, TA ↓, LDH ↓, CK ↓, calcium ↓
Doxycycline	Rat [29]	Heart	HMP	Apoptosis ↓, MMP-2 ↓, H_2_O_2_ ↓, pAkt/Akt ↑

**(c):** 

Treatment	Subject	Organ	Model or disease	Effects
*Ischemic postconditioning*				
RIPoC	Human [56]	Kidney	KT	Cr ↓, pathology (—), GFR ↑, uNGAL ↓
	Human [57]	Liver	LiT	No beneficial effect
	Human [58]	Kidney	KT	No beneficial effect
LIPoC	Canine [61]	Kidney	KT	MDA ↓, MPO ↓, SOD ↑, apoptosis indices ↓, Cr ↓, BUN ↓, Ccr ↑
	Human [62]	Kidney	KT	Safe but no beneficial effect

ACR: acute cellular rejection; ALAS-1: 5-aminolevulinate synthase; ALT: alanine aminotransferase; AST: aminotransferase; ATP: adenosine triphosphate; BSP: bromosulfophthalein; BUN: blood urea nitrogen; Ccr: creatinine clearance; cGMP: cyclic guanosine monophosphate; Cox-2: cyclooxygenase-2; Cr: creatinine; DGF: delayed graft function; eNOS: endothelial nitric oxide synthase; ET-1: endothelin-1; GFR: glomerular filtration rate; GLDH: glutamate dehydrogenase; GST: glutathione S transferase; HA: hyaluronic acid; HBV: hepatitis B virus; HMP: hypothermic machine perfusion; HSP: heat shock protein; HT: heart transplant; HUVECs: human umbilical endothelial cells; IAT: intestinal allotransplantation; iNOS: inducible nitric oxide synthase; I/R: ischemia and reperfusion; KT: kidney transplantation; LDH: lactate dehydrogenase; LDL: low density lipoprotein; LIPoC: local ischemic postconditioning; LiR: liver resection; LiT: liver transplantation; LPS: lipopolysaccharide; LuT: lung transplantation; MDA: malondialdehyde; MPO: myeloperoxidase; MV: mechanical ventilation; NOS: nitric oxide synthase; NPSH: nonprotein thiols; pAkt: phosphorylated Akt; PARP: poly(ADP-ribose) polymerase; PLiT: partial liver transplantation; PNF: primary nonfunction; PT: pancreas transplantation; PVF: portal venous flow; PVP: portal venous pressure; RIPoC: remote ischemic postconditioning; TA: total antioxidants; TBiL: total bilirubin; TNF: tumor necrosis factor; TLR-4: toll-like receptor-4; T2DM: type 2 diabetes mellitus; SCS: static cold storage; SOD: superoxide dismutase; uNGAL: urine neutrophil gelatinase-associated lipocalin; VEGF: vascular endothelial growth factor; W/D: wet-to-dry; 4-HNE: 4-hydroxyalkenals; 8-OHdG: 8-hydroxy-2-deoxyguanosine.

## Abbreviations

AA: Ascorbic acid
ACE: Angiotensin-converting enzyme
AOE: Antioxidant enzymes
CABG: Coronary artery bypass grafting
CO: Carbon monoxide
CYPs: Cytochrome P450
DGF: Delayed graft function
DOX: Doxycycline
HES: Hydroxyethyl starch
HMP: Hypothermic machine perfusion
HO-1: Hemeoxygenase-1
HRUW: Hydrogen-rich UW solution
HRW: Hydrogen-rich water
H_2_S: Hydrogen sulfide
IGL-1: Institute Georges Lopez-1
iNOS: Inducible nitric oxide synthase
I/R: Ischemia and reperfusion
IRI: Ischemia/reperfusion injury
LIPC: Local ischemic preconditioning
LIPoC: Local ischemic postconditioning
LPO: Lipid peroxidation
LT: Liver transplantation
MDA: Malondialdehyde
MMPs: Matrix metalloproteinases
MP: Machine perfusion
MPT: Mitochondrial permeability transition
MV: Mechanical ventilation
NHBD: Non-heart-beating donors
NMP: Normothermic machine perfusion
NO: Nitric oxide
PEG: Polyethylene glycol
PNF: Primary nonfunction
PECAM-1: Platelet/endothelial cell adhesion molecule-1
RCT: Randomized clinical trial
RIPoC: Remote ischemic postconditioning
SCOT: Solution de Conservation des Organes et des Tissus
SCS: Static cold storage
SNMP: Subnormothermic machine perfusion
SOD: Superoxide dismutase
VILI: Ventilator-induced lung injury
VSOP: Venous systemic oxygen persufflation.
